# A Satellite–Ground Hybrid Approach: Relative Risks for Exposures to PM_2.5_ Estimated from a Combination of Data Sources

**DOI:** 10.1289/ehp.125-A73

**Published:** 2017-03-31

**Authors:** Nate Seltenrich

**Affiliations:** Nate Seltenrich covers science and the environment from Petaluma, CA. His work has appeared in *High Country News*, *Sierra*, *Yale Environment 360*, *Earth Island Journal*, and other regional and national publications.

Satellite instruments offer researchers powerful new perspectives and data sources for studying the environment. A new study used associations between fine particulate matter (PM_2.5_) and mortality from circulatory diseases as a test scenario to explore how exposure estimates derived from remote sensing alone compare with those produced by a combination of satellite- and ground-based data.^1^ The findings showed associations between PM_2.5_ and mortality regardless of the method used, but specific relative risk estimates varied widely, with hybrid models generally predicting the strongest associations.

**Figure:**
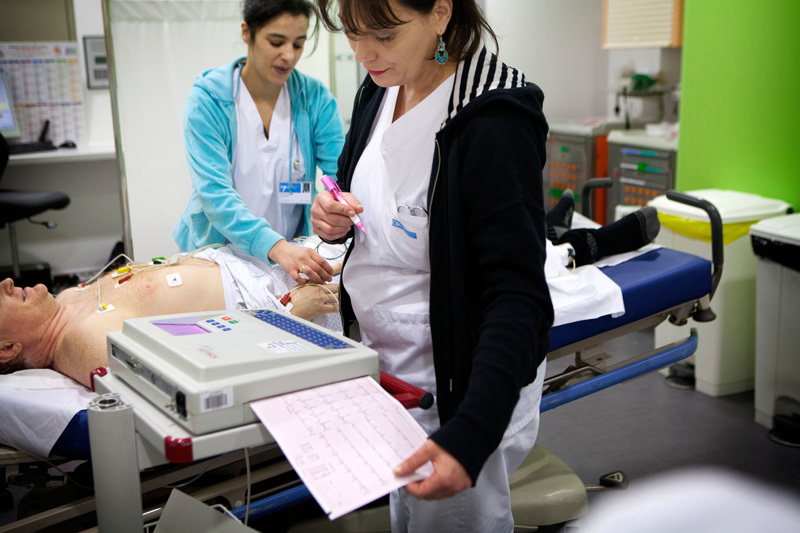
Fine particulate matter has been strongly implicated in cardiovascular problems including heart attacks and strokes. Neither remote sensing nor ground-based monitors alone can fully capture a population’s exposures to air pollutants, but the combined use of these technologies may paint a more complete picture. © BSIP SA/Alamy Stock Photo

NASA’s Terra satellite carries a variety of instruments for observing our planet’s atmosphere and surface from a height of 443 miles.^2^ Remote sensing is best at detecting differences on a broad scale, such as changes in air quality over time or across geographic regions, as well as pollution sources and sinks, says professor Yang Liu of Emory University, who was not involved in the study.^3^ ^,^ ^4^ Ground-based monitors, on the other hand, directly measure air pollutants in their vicinity but may not be accurate for estimating exposures to individuals who are not at the same location. In addition, ground-based monitors are not available in many locations.

As researchers worldwide have enjoyed increasing access to high-resolution air quality and atmospheric data obtained through remote sensing, they have developed ways of using the data to more accurately estimate human exposures to air pollution. Recent trends and advances call for a closer investigation into similarities and differences among satellite- and ground-based models, says lead author Michael Jerrett, chair of the Department of Environmental Health Sciences at the University of California, Los Angeles, Fielding School of Public Health.

“We felt that with the proliferation of models and different approaches, we wanted to be able to understand what this would mean for exposure estimates and therefore for policy and global burden-of-disease calculations,” Jerrett says.

Jerrett and colleagues selected a representative sample of seven such models for comparison, to see how differences among them would influence estimates of health effects on U.S. residents enrolled in the American Cancer Society Cancer Prevention Study II.^5^ Some of the models relied exclusively on satellite data, some on ground-based monitors, and others on a combination of data sources.

The researchers geocoded the residences of 668,629 participants for the years 2002–2004 and estimated individual exposures to PM_2.5_ using the methods dictated by the various models. The researchers then estimated relative risk of mortality from circulatory diseases in association with the PM_2.5_ exposures predicted by each approach.^6^

They discovered that associations between PM_2.5_ and mortality were significant for all seven models—a meaningful though tangential result, Jerrett says. However, the models differed considerably in their estimate of the magnitude of that association. The smallest estimate of relative risk came from the two remote-sensing models without ground data, and the largest from the model with the most extensive ground data: a blend of air quality measurements from monitors, land-use modeling, and traffic density data.

“The key finding of the paper is that when you don’t have any ground data represented in remote-sensing models, they do tend to produce risk estimates that appear to be biased toward the null,” Jerrett explains—in other words, the actual relative risk may be underestimated. This has important implications for researchers investigating health effects of air pollution in parts of the world without sufficient ground-based monitors to fill in the complementary remote-sensing data. The findings support the conclusion that whenever possible, hybrid models and combinations of multiple models should be used for maximum coverage and accuracy.

Coauthors Randall Martin and Aaron von Donkelaar of Dalhousie University, who developed the study’s remote-sensing exposure models, agree that satellites should not stand alone. “There are a variety of information sources from which to obtain and learn about PM_2.5_, and we have often promoted the use of as many of those as possible,” Martin says. “The results provide motivation to pay close attention to how ground data are fused with remote-sensing estimates. They are independent measurements that have arisen for largely different reasons, but there’s synergy in combining both information sources to learn what we can about fine particulate matter.”

Beyond clarifying this relationship, the study should have even more tangible, direct benefits for the field, says professor Julian Marshall of the University of Washington, who was not affiliated with the study. Marshall praised the study for its use of multiple rigorous estimates of exposure. “The authors went to significant lengths to get many robust exposure estimates,” he says. “Many of the methods used here are publicly available and could be useful for many, many cohorts. That opens up many new doors.”

## References

[1] Jerrett M Comparing the health effects of ambient particulate matter estimated using ground-based versus remote sensing exposure estimates. Environ Health Perspect 125 4 552 559 2017, doi:10.1289/EHP575 27611476 PMC5382001

[2] NASA Terra Spacecraft [website].. http://www.nasa.gov/mission_pages/terra/spacecraft/index.html.

[3] van Donkelaar A Global estimates of ambient fine particulate matter concentrations from satellite-based aerosol optical depth: development and application. Environ Health Perspect 118 6 847 855 2010, doi:10.1289/ehp.0901623 20519161 PMC2898863

[4] Geddes JA Long-term trends worldwide in ambient NO2 concentrations inferred from satellite observations. Environ Health Perspect 124 3 281 289 2016, doi:10.1289/ehp.1409567 26241114 PMC4786989

[5] ACS Cancer Prevention Study II (CPS II) [website].. http://www.cancer.org/research/researchtopreventcancer/currentcancerpreventionstudies/cancer-prevention-study.

[6] MedCalc Cox proportional-hazards regression [website].. https://www.medcalc.org/manual/cox_proportional_hazards.php.

